# Insights into Polyphenol–Lipid Interactions: Chemical Methods, Molecular Aspects and Their Effects on Membrane Structures

**DOI:** 10.3390/plants11141809

**Published:** 2022-07-08

**Authors:** Maarit Karonen

**Affiliations:** Natural Chemistry Research Group, Department of Chemistry, University of Turku, 20014 Turku, Finland; maarit.karonen@utu.fi; Tel.: +358-29-4503179

**Keywords:** bioactivity, flavonoid, lipid bilayer, lipid vesicles, macromolecule, phenolics, structure-activity, tannin

## Abstract

Plant polyphenols have many potential applications, for example, in the fields of chemical ecology and human and animal health and nutrition. These biological benefits are related to their bioavailability, bioaccessibility and interactions with other biomolecules, such as proteins, lipids, fibers and amino acids. Polyphenol–protein interactions are well-studied, but less is known about their interactions with lipids and cell membranes. However, the affinity of polyphenols for lipid bilayers partially determines their biological activity and is also important from the usability perspective. The polyphenol–lipid interactions can be studied with several chemical tools including, among others, partition coefficient measurements, calorimetric methods, spectroscopic techniques and molecular dynamics simulation. Polyphenols can variably interact with and penetrate lipid bilayers depending on the structures and concentrations of the polyphenols, the compositions of the lipids and the ambient conditions and factors. Polyphenol penetrating the lipid bilayer can perturb and cause changes in its structure and biophysical properties. The current studies have used structurally different polyphenols, diverse model lipids and various measuring techniques. This approach provides detailed information on polyphenol–lipid interactions, but there is much variability, and the results may even be contradictory, for example, in relation to the locations and orientations of the polyphenols in the lipid bilayers. Nevertheless, by using well-characterized model polyphenols and lipids systematically and combining the results obtained with several techniques within a study, it is possible to create a good overall picture of these fascinating interactions.

## 1. Introduction to Polyphenol–Lipid Interactions

**Figure 1:**
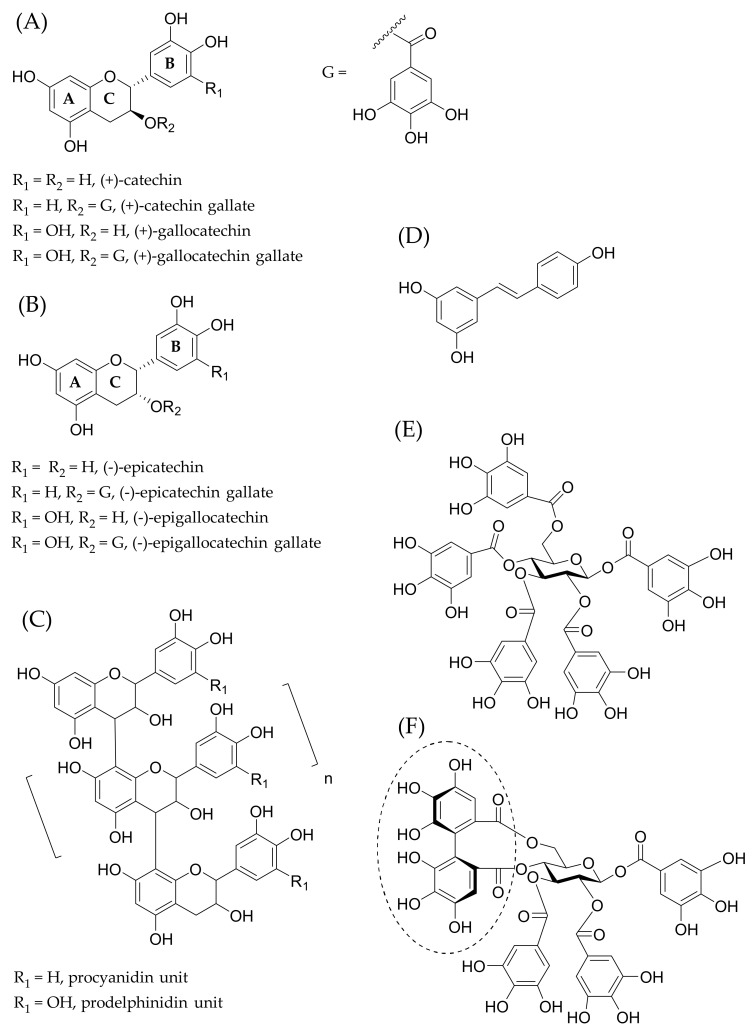
Examples of polyphenols having affinities to lipids: (**A**) flavan-3-ols with 2*R*,3*S* stereochemistry, (**B**) flavan-3-ols with 2*R*,3*R* stereochemistry, (**C**) B-type proanthocyanidins linked by a C4–C8 bond, (**D**) resveratrol, (**E**) pentagalloylglucose and (**F**) tellimagrandin II (the characteristic hexahydroxydiphenoyl group for ellagitannins is highlighted).

Polyphenols are plant specialized metabolites (also known as secondary metabolites) having antioxidant, anticarcinogenic, antitumoral, antiviral, antimicrobial, anti-inflammatory and anti-fibrillogenic properties; they derive exclusively from the shikimate-derived phenylpropanoid or polyketide pathways, feature more than one phenolic ring and are devoid of any nitrogen-based functional group [1]. Polyphenols can be classified into flavonoids, stilbenes, lignans and tannins including phlorotannins, proanthocyanidins (PAs) and hydrolysable tannins (HTs), which can be further divided into simple gallic acid derivatives, gallotannins and ellagitannins (Figure 1).

Plant polyphenols have been intensively investigated due to their potential positive health effects and the possibility of using them in animal nutrition and health [1,2,3,4,5,6,7,8,9,10]. The biological activity of polyphenols is highly structure-dependent, and in biological applications, the bioavailability and bioaccessibility of polyphenols and their interactions with other compounds present in the plant matrices play an important role [2,11,12].

Polyphenols are known to have interactions with all macromolecules: proteins, carbohydrates, lipids and amino acids. The polyphenol–protein interactions, especially tannin–protein interactions, are well-known [13]. The interactions of polyphenols with the other macromolecules have not been studied as comprehensively, but for example, polyphenols do interact with the various cell components such as pectin, cellulose and fibers [11,14,15,16] and also with the nucleic acids [17]. These interactions between polyphenols and macromolecules are complex and also affected by micronutrients such as minerals, vitamins and organic pigments as well as environmental factors such as pH and temperature [12,18]. The affinity of polyphenols for lipid bilayers partially determines their biological activity in vitro [19] and is also important from the medical and dietary points of view [20]. For example, flavonoids are known to regulate cell metabolism either by intercalating the membrane lipid or by controlling cell signal pathways [21]. Polyphenols can have direct interactions with simple food lipid ingredients causing a decrease in the their absorption or the reduced occurrence of harmful lipid oxidation products [2]. However, these aspects are not discussed in this review; instead the focus is on the complex lipids and lipid membranes.

**Figure 2:**
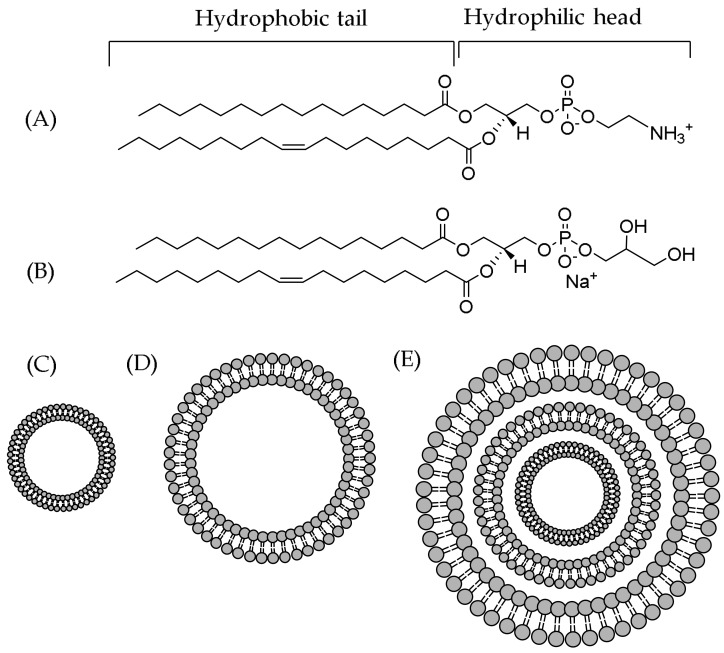
Examples of the model phospholipids used for the biophysical and interaction experiments: (**A**) 1-palmitoyl-2-oleoyl-sn-glycero-3-phosphocholine (POPC) and (**B**) 1-palmitoyl-2-oleoyl-sn-glycero-3-phosphatidylglycerol (POPG), and the classification of liposomes based on their lamellarity: (**C**) small unilamellar vesicle (around 20–100 nm) and (**D**) large unilamellar vesicle (around 100–250 nm) consisting of a single phospholipid bilayer, and (**E**) multilamellar vesicle (around 1–5 µm) consisting of many phospholipid bilayers. The vesicles are not on the same scale but are structural examples.

Lipids are molecules that are classically thought to comprise a hydrophilic head and two hydrophobic tails [22]. However, many lipid classes do not follow this definition, and therefore, based on their structural, biosynthetic and chemical properties such as hydrophobic and hydrophilic elements, a comprehensive classification system has been suggested dividing them into eight different categories [23]. These widely accepted lipid categories include fatty acyls, glycerolipids, glycerophospholipids, sphingolipids, sterol lipids, prenol lipids, saccharolipids and polyketides [23,24,25]. In aqueous environments, the lipids self-assemble into lipid bilayers, i.e., they form a 2-dimensional biomolecular sheet consisting of 2 layers of lipid molecules aligned in a nearly parallel manner to form a hydrocarbon core; hydrophilic headgroups are layered on both sides of the hydrocarbon core [26]. The model lipids used for cell membranes are complex, and lipid compositions vary: The lipid composition can be single, binary or multiple; the shape can be uni- or multilamellar; and the size can vary from small to large or giant [27,28]. Unilamellar vesicles, that is small or sonicated unilamellar vesicles (SUV), and large unilamellar vesicles (LUVs) are prepared from multilamellar vesicles (MLVs), for example by extrusion (Figure 2).

The size of vesicles can be confirmed by dynamic light scattering (DLS) analysis [20,29] or by microscopic techniques such as transmission electron microscopy (TEM) or scanning electron microscopy (SEM) [30,31]. Model lipids include various sizes and different degrees of saturation in lipid tails, different charges on lipid heads (although in biological membranes, most of the lipids are zwitterionic and exist without an overall charge [32]) and mixtures of different lipids. These models can mimic the bacteria cell membrane; the microorganism cell membrane system, such as the phospholipid extract of *E*. *coli* [33,34]; the yeast cell membrane [33]; egg L-α- phosphatidylcholine (EPC); the most abundant lipids in cell membranes [20,35] and cholesterol and sphingomyelin, the essential components of the human cell membranes [35,36]. Liposomes can be prepared by lipid film hydration [35,37], and their preparation was nicely summarized by Šturm and Ulrih [38].

As mentioned above, the interactions between polyphenols, especially tannins, and proteins are very well studied, and the interactions of polyphenols with membrane lipids in vitro and the related challenges are also known [13,27]; however, few studies focus on the exact interactions between polyphenols and lipids. Chemical tools including spectroscopic techniques and molecular dynamics simulations offer the possibility of observing the interactions between polyphenols and lipids in detail and at a molecular level. In these interactions, both the structural features and concentrations of the polyphenols and lipids have effects as discussed above. Here, the focus is mainly on different chemical techniques that can be used to evaluate the interactions between polyphenols and lipid membranes, followed by a brief discussion of results that can be and have been obtained by these tools. The current knowledge is summarized: what is already known regarding the structural features of polyphenols, their location in the lipid membranes and the changes they might cause to the lipids; the remaining challenges and the extent of the effects of polyphenols are still ambiguous.

## 2. Methods

The methods used to study polyphenol–lipid interactions vary from simple partition coefficient measurements to more detailed calorimetric, spectroscopic and computational techniques. There are also less commonly used techniques, such as noncovalent immobilized artificial membrane chromatography [39] and electrochemical measurements [40]. All methods have their own advantages and challenges, and therefore, the interaction is typically evaluated using a complementary set of different techniques and diverse polyphenols and lipids. This approach provides detailed and reliable information on polyphenol–lipid interactions, but it also poses challenges for comparing results obtained with different methods, different model substances and different conditions.

### 2.1. Partition Coefficient Measurements

Partition coefficient measurements in biphasic solvent systems, such as *n*-octanol-water (*K_ow_*), have been traditionally used to study the physicochemical properties of polyphenols [41]. Hydrophobicity is one of the essential properties that affect how the compound interacts with lipids or permeates cell membranes. The most common solvent pair in partition coefficient measurements is octanol–water; however, also alkane–water and heptane–ethylene glycol have been used [42]. The same partitioning takes place in biological systems in vivo, for example, when different compounds are transported into cells [42]. The compounds with high octanol solubilities, i.e., higher *K_ow_* coefficients, tend to be more easily absorbed into tissues [43,44].

#### 2.1.1. Partition Coefficients by Octanol-Water

The traditional *K_ow_* method is relatively simple as the polyphenol studied is dissolved in an octanol–water system and allowed to equilibrate, after which both phases are analyzed by high- or ultrahigh-performance liquid chromatography with UV detection [43,44,45]. *K_ow_* coefficients can be calculated from the ratio of the peak area in the water-saturated octanol phase to the peak area in the octanol-saturated water phase. In some cases, *K_ow_* measurements have been performed so that the concentration of the analyte was analyzed in only one of the phases (either octanol or water) and in the initial stock solution before the analyte concentration in the unanalyzed phase was calculated as their difference. This approach might ignore the recovery of the analyte due to poor solubility or other issues. Therefore, the initial sample and the samples from both the water and octanol phases should be analyzed in order to determine the recovery rate of the polyphenol studied and transition to both phases accurately [43,45]. *K_ow_* can also be measured using simple ^1^H nuclear magnetic resonance (NMR), where the partitioning between water and octanol is performed in an NMR tube and the aqueous phase is then analyzed by NMR provided that the analyte is sufficiently soluble in water [46]. In addition to the experimental methods, the partition coefficients as log *p* values can be determined by in silico predictions using structure-based computational tools such as AlogP, Molinspiration and COSMOtherm [47]. These methods use topological descriptors or the fragmentation of the molecules to functional groups and atoms and have been shown to progenerate similar trends to the experimentally determined values for the polyphenols in milk thistle seeds [47]. In general, these methods work well, especially when the molecules studied are similar to the training set; however, there can be differences between the predicted and experimental values [47].

Published *K_ow_* coefficients seem to vary between studies [43]. This is most probably due to the different methods used. However, there are clear similar trends detected in different studies. For example, the galloyl glucoses and gallotannins are more hydrophobic than the ellagitannins, and there is a clear relationship between the number of galloyl groups present in galloyl glucoses and gallotannins and their partition coefficients between octanol and water [45,48]. In general, nonpolar flavonoid aglycones have higher *K_ow_* than polar flavonoid glycosides, and PA gallates are more hydrophobic than PAs [43,44]. The presence and number of free galloyl groups seem to be the most important factor determining the hydrophobicity within a polyphenol class [44,45,48]. However, the lipophilicity of the compound does not solely predict its antimicrobial activity or cell membrane permeability [42,49]. The information must be linked, for example, with forces such as hydrogen bonding and hydrophobic effect [42].

#### 2.1.2. Partition Coefficients in Membrane Models

The partition coefficients of polyphenols can also be evaluated in membrane models by determining the partition of polyphenols between the model lipid membranes and the aqueous phase as *K_p_* [37,50,51]. The model lipids can be, for example, dimyristoylphosphatidylcholine (DMPC), dipalmitoylphosphatidylcholine (DPPC), EPC, cholesterol, linoleic acid and sphingomyelin or their mixtures [37,50]. The partition coefficients of resveratrol between membrane mimetic LUV suspensions and aqueous buffer solution have been determined by derivative spectroscopy [37]. In this method, polyphenol in buffer solution is added to lipid suspensions and incubated with agitation. The reference is prepared similarly but without the polyphenol. After the incubation, the absorption spectra of both the sample and reference solutions are measured and the results processed mathematically [37,52]. In addition, polyphenol–lipid partition with MLVs has been determined by estimating the polyphenol concentration in the supernatant with the Folin method [50]. In this model, the percentage of polyphenol partition is calculated simply as [50]:

partition-% = ([polyphenol]_added_–[polyphenol]_supernatant_)/[polyphenol]_added_ × 100 (1)

The octanol–water and liposome–water partitioning models have been compared using 66 drugs [53]. For the neutral drugs, both models produced similar results, whereas for the charged drugs (positively, negatively or partially ionized/zwitterionic), the liposomal model produced more appropriate results than the traditional *K_ow_* model [53]. Similarly to the computational tools of determining octanol-water partition coefficients, the lipid-water partitioning in membrane models has been predicted by computational methods, for example, using dioleoylphosphatidylcholine (DOPC), ceramide (CER) or a mixture of CER, lignoceric acid and cholesterol as models by COSMOmic [47]. These calculations also included free energy profiles, permeability and a penetration barrier (kcal/mol).

### 2.2. Calorimetric Techniques

**Figure 3:**
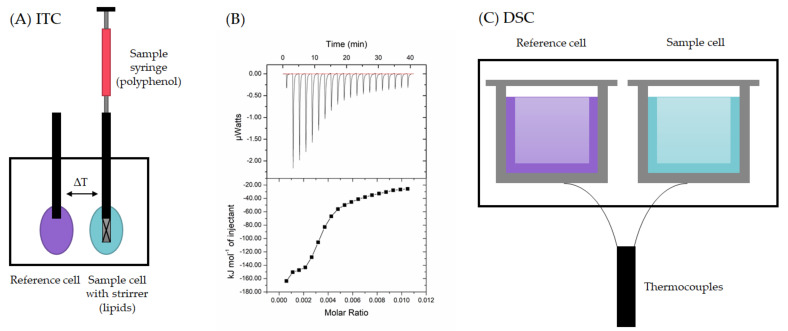
(**A**) Isothermal titration calorimeter (ITC), (**B**) raw ITC data (upper panel) and the thermogram (lower panel) of ellagitannin rugosin G titrated into lipid vesicles of *Escherichia coli* (control titration of ellagitannin into buffer subtracted), (**C**) differential scanning calorimeter (DSC) with crucible cells for sample and reference [29,63].

The thermodynamics of the polyphenol–lipid interaction can be studied and the related thermodynamic binding parameters determined by calorimetric techniques. Isothermal titration calorimetry (ITC, Figure 3A) is a technique that enables the direct measurement of the energetics of the interactions and the stability, strength, specificity and stoichiometry of the interacting systems [54]. The method is based on the fact that the interaction between a macromolecule (here the lipid vesicle) and its ligand (here the polyphenol) is accompanied by a heat effect. ITC can be utilized to determine the stoichiometry of the binding, *n*; the binding constant *K_a_*; and the enthalpy of the binding *ΔH*, thereby also allowing for determining the entropy *ΔS*, the Gibbs free energy change *ΔG*, and the heat capacity change *ΔCp* [54,55]. ITC has been quite intensively applied to study the interactions of peptides or proteins with model and natural membrane phospholipids [56,57,58,59,60,61,62]. However, so far, there seems to be only one ITC study on polyphenol–lipid interactions focusing on the interactions between HTs and lipid vesicles prepared from the total phospholipid extract of *Escherichia coli* [29]. In this study, the interactions between tannins and lipids were found to be exothermic (heat released), and the strongest interaction was observed for rugosin G up to – 160 kJ/mol when 0.1 mM tannin solution was titrated into 2 mM lipid solution as shown in Figure 3B [29].

In a typical ITC measurement, the lipid solution is placed in the sample cell of the calorimeter, and the polyphenol solution is titrated into the lipid incrementally [29,54,64]. The control measurements are important for excluding the other heats present. These experiments typically include the titration of the polyphenol solution into buffer (the heat of the dilution of the ligand), the titration of the buffer into the lipid solution (the heat of the dilution of the macromolecule) and the titration of buffer into buffer (instrument blank) [65]. Usually, the heat of the dilution of the polyphenol is significant. Tannins especially tend to self-associate into aggregates in concentrated aqueous solution and then undergo an endothermic process of deaggregation when injected from the syringe into buffer [29,66,67,68,69,70,71]. Therefore, these control data need to be subtracted from the sample data. The other control measurements, buffer into macromolecule and buffer into buffer, typically give negligible heats [29,60,71]. In general, ITC is easy to use, and reasonable heats of injections and sigmoidal thermograms enable fluent fitting and the determination of *n*, *K_a_* and *ΔH*. However, these are not always easy to obtain, and experimental compromises are needed that can cause challenges in later data interpretation and fits. In any case, for correct results, the concentrations of ligand and macromolecules need to be accurately known [65], although there is still a possibility of statistical errors in the processing of the ITC data [64].

Differential scanning calorimetry (DSC), like ITC, is used for the identification of the physical and chemical changes and thermal transitions of polymeric molecules, such as for peptides and lipids [61]. Thermoanalytical DSC measures the heat capacity of a solution as a function of temperature: In DSC, the perturbation is a change in temperature of the sample, while in ITC, it is the injection of sample solution [63]. In DSC, the sample is placed in the sample cell (Figure 3C), and the DSC produces information on the temperature and heat flow through the sample cell while the samples are exposed to a temperature program [72]. In practice, the technique measures the differences in heat flow rates, i.e., the heat flow of the reference cell is subtracted from the heat flow of the sample cell. The instrument can be a heat-flux DSC, where suitable calorimetric calibration is needed to obtain a direct measure of the temperature difference between the cells, or a power compensation DSC, where the difference in power supplied to the cells to maintain them at the same temperature is measured [72]. The reference material should be inert over the temperature range studied. The reference can be, for example pure solvent or buffer solution, the sample can be diluted with the reference, and it is also possible to use an empty cell as a reference [63,72]. In a common DSC measurement, both cells are loaded (or the reference is left empty), equilibrated and scanned with heat, cool and heat cycles, for example at scan rate of 1 °C/min from 10 °C to 60 °C [73] or of 2 °C/min from 20 °C to 50 °C [20]. DSC is easy to use and widely applied, but attention needs to be paid to the liposome concentrations and the final concentrations of organic solvents [38]. In general, DSC is a sensitive technique for studying phospholipids such as phosphatidylcholines, but for example, studies on cholesterol have been performed using lipid mixtures with low concentrations (<10%) of cholesterol because the high concentrations notably affected the phase transition; they broadened the endotherm and interfered with the L_c_/L_α_ transitions [74,75,76], making DSC more suitable for cholesterol-depleted than for cholesterol-rich membranes.

DSC can notably be used to determine the phase transition temperature T_m_ and the transition enthalpy *ΔH* when the lipid bilayer undergoes thermally induced phase transitions [26]. In the analysis of the effects of the polyphenols from blueberry fruits on lipid model membranes, an empty crucible was used as reference, and indium was used for the temperature calibration [20]. In this study, the phenolic extracts did not cause significant changes in the temperatures of the pretransition or the main transition of the DPPC lipid membrane even though they were found to modify the hydrophilic part of the membrane through electrostatic interactions with the polar heads of the lipids. In studies of the interactions between chemically distinct phenolics, for instance the specialized metabolites resveratrol and quercetin in addition to raloxifene, bisphenol A and garcinol containing a phenolic ring, and DPPC bilayers, it was noticed that hydrophobic raloxine increased the melting temperature (T_m_), whereas the other compounds caused a slight reduction in T_m_ [73]. In the analysis of the interaction between citrus flavonoids and DMPC lipid membranes, DSC revealed that naringin significantly influenced the cooperativity and thermotropic phase behavior of the model lipid membrane in comparison with hesperidin [77]. In general, ITC and DSC could be used as complementary tools to investigate polyphenol–lipid interactions as ITC provides the global thermodynamic parameters of the reactions and DSC the detection of transitions, for example, from the conformational changes of the component molecules during the association [55].

### 2.3. Spectroscopic Techniques

Spectroscopic techniques can be used to obtain detailed structural information but also to study interactions at the molecular scale. There are many spectroscopic techniques available, but in this section, the most used techniques related to polyphenol–lipid interactions are discussed.

#### 2.3.1. Fourier Transform Infrared Spectroscopy

The penetration and distribution of polyphenols into lipid bilayers and the related forces can be studied by the changes in infrared absorption spectra obtained by Fourier transform infrared (FTIR) spectrophotometer, which can provide spectra in the common range of 4000 to 400 cm^−1^ for both liquid and solid samples [78]. For example, the incorporation of apigenin into DPPC liposomes has been shown to occur via hydrogen bonding between its OH groups and the polar head groups of the lipid as the spectral region corresponding to the C-O-P-O-C vibration (1054 cm^–1^) is strongly affected [79].

The best method for solid samples is to use an attenuated total reflectance (ATR) accessory, which has facilitated the IR analysis of solids and today is offered along with the typical transmittance module [78]. With the ATR accessory, a small amount of a liquid or solid sample is placed directly on the diamond or crystal without previous preparation. ATR–FTIR can measure the changes in the hydrophilic part of the membrane, i.e., the degree of the membrane hydration modified with the polyphenols [20]. Liposomes are shaken with polyphenols and measured using ATR–FTIR with ZnSe crystal at room temperature [20]. After the measurements, the noise is filtered from the spectrum, and then the spectrum of the buffer solution is subtracted in order to remove the OH band of water; finally, the baseline is corrected. The studies take into account, for example, four different bands: the symmetric and antisymmetric C-H stretching vibrations of CH_2_ and CH_3_ of alkyl groups in the range of 3000–2800 cm^−1^, the vibrations of the carbonyl groups (C=O) in the range of 1780–1700, the vibrations of the phosphate groups (PO_2_) in the range of 1300–1200 cm^–1^, and the vibrations of choline groups (N-CH_3_) in the range of 1000–900 cm^–1^ [20,79]. ATR–FTIR spectroscopy has been used, for example, to study the DMPC membrane effects of hesperinin and naringin localized in the hydrophobic and hydrophilic ranges of lipid membranes [77]. In this study, both hesperinin and naringin were found to increase the bandwidths of CH_2_ antisymmetric stretching vibration, indicating an increase in the fluidity of the membranes. The advantages of FTIR are the minimal sample preparation needed and the good reproducibility of the measurements. Most commonly, ATR–FTIR is applied for MLVs [20,62,77], which might cause differences when comparing the results with results obtained with other techniques and other lipid membrane models, for example, as mentioned en passant for the interactions of procyanidin B_3_ with DMPC membranes studied by fluorescence, DSC and FTIR obtained using an oriented flat multilamellar membrane and liposomes [80].

#### 2.3.2. Fluorescence Anisotropy Techniques

Another spectroscopic technique used to study polyphenol–lipid interactions and the locations of polyphenols is fluorescence anisotropy, which utilizes a polarized fluorescence excitation light beam to excite fluorescent probes embedded in the lipid regions of the membrane samples [81,82]. The effects of polyphenols on the fluidity of different lipid phases can be evaluated on the basis of the fluorescence anisotropy, which is measured using a fluorescent probe that locates itself in the lipid bilayers. There are many fluorescent probes available for studying the polyphenol–lipid interactions. The main differences in these probes are related to their positioning within the lipid bilayer depending on their chemical structures and properties. The fluorescent probe can be located at the hydrophilic region of the lipid bilayer, such as 1-(4-trimethyl-ammonium phenyl)-6-phenyl-1,3,5-hexatriene (TMA-DPH) [83,84,85], 2-(9-anthroyloxy)stearic acid (2-AS) [85], 5-doxyl-stearic acid (5-NS) [86], 6-dodecanoyl-2-dimethylaminonaphthalene (laurdan) [20] and *N*,*N*-dimethyl-6-propionyl-2-naphthylamine (prodan) [20]. The fluorescent probe can also be located at the hydrophobic region of the lipid bilayer, such are, for example, 1,6- diphenyl-1,3,5-hexatriene (DPH) [83,84,86], 16-doxyl-stearic acid (16-NS) [86], 9-(9-anthroyloxy)stearic acid (9-AS) [85] and trans-parinaric acid (t-PnA) [87]. Typically, the studies have been carried out using more than one fluorescent probe, for example, both DPH and TMA–DPH [83,84]. Pruchnik et al. used both the hydrophilic fluorescent probes laurdan and prodan and hydrophobic DPH probe; the laurdan and prodan probes allow for the investigation of the packing order of the hydrophilic part of the lipid bilayer, whereas the steady-state anisotropy of DPH is related to the restricted rotational motions due to the hydrocarbon chain packing order. Thus, the decrease in this parameter reflects the perturbation in the hydrophobic region of the lipid bilayer [20]. It was shown that by comparing the results of fluorescence emission with the anisotropy measurements, the same fluorescent probe, in this case laurdan, could reflect the changes in membrane fluidity (laurdan aniosotropy) and in phospholipid order (laurdan generalized polarization) as the fluorescence anisotropy is directly correlating with the membrane microviscosity and consequently inversely correlating with its fluidity [88,89]. In general, the relevant parameter is the steady-state fluorescence anisotropy, and the reduction in the anisotropic emission indicates membrane rigidity caused by the restriction of the acyl chain movements, whereas the increase in the anisotropic emission indicates enhanced membrane fluidity caused by the molecular interactions [81,82]. In the steady-state fluorescence measurement, a fluorescent probe, for example laurdan dissolved in DMSO [89], is added to lipid vesicles placed in the cuvette of the spectrofluorometer. Then to the stained liposomes, well-known concentrations of polyphenols are added in order to measure the effects of the polyphenols on the fluorescence emission of the probe. The fluorescence emission spectra are recorded in the range of 400-555 nm using excitation at 364 nm with and without polarizers in the excitation and emission beams [89]. The blanks without the probe are needed to subtract the background fluorescence. The measurements can be performed at different temperatures above and below the main phase transition of the lipid [20]. In general, the advantages of fluorescence anisotropy are its high sensitivity, enabling the use of low sample concentrations, and the possibility of using a broad range of experimental conditions [90,91], but in the analysis, attention must be paid to the size of the liposomes. The accuracy of the measurements depends on the quality and alignment of the polarizers, i.e., on the accuracy of the instrument [92,93].

#### 2.3.3. NMR Spectroscopy

NMR spectroscopic techniques are powerful and versatile methods for studying polyphenol–lipid interactions. Solid-state NMR spectroscopy can be used to study the structural and dynamic properties of polyphenols incorporating into membrane lipid bilayers and to elucidate the locations and interactions of small molecules in lipid bilayers [94,95,96]. These methods include solid-state ^13^C NMR, ^31^P and ^2^H spectroscopy utilizing a cross-polarization magic angle spinning (CP-MAS) probe. The solid-state ^31^P NMR tells about the interactions between polyphenols and the head group region of lipids. ^31^P has a wide chemical shift range, making it a sensitive probe for changes in its magnetic environment; therefore, the lipid phosphate group expresses well the changes in the water–lipid surfaces [96]. ^2^H is another interesting nucleus in biological solid-state NMR. In solid-state ^2^H NMR, the long lipid chain can be perdeuterated to report on the hydrophobic interior [95,96]. The theoretical basis for deuterated lipid systems has been nicely described by Furlan et al. [95].

**Figure 4:**
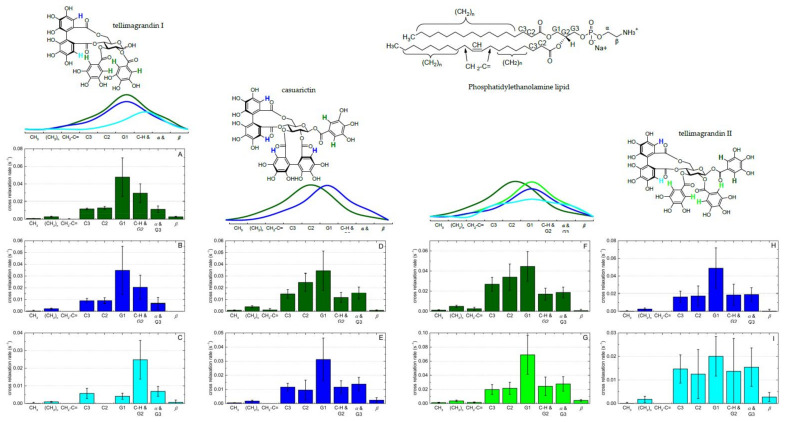
The cross-relaxation rates (s^–1^) between different protons of ellagitannins and lipid protons of *Escherichia coli* obtained from HR–MAS NMR by nuclear Overhauser effect measurement with a mixing time of 0.1 ms [34]. The cross-relaxation rates provide the distribution functions of the (**A**) galloyl group protons attached to glucose O2 and O3, (**B**) hexahydroxydiphenoyl (HHDP) protons attached to glucose O6, (**C**) HHDP protons attached to glucose O4, (**D**) galloyl group protons attached to glucose O1, (**E**) HHDP protons attached to glucose O2, O3, O4 and O6, (**F**) galloyl group protons attached to glucose O1, (**G**) galloyl group protons attached to glucose O2 and O3, (**H**) HHDP protons attached to glucose O6, and (**I**) HHDP protons attached to glucose O4. Above the cross-relaxation rates, sketches of the approximate membrane location of ellagitannins are shown.

High-resolution magic angle spinning (HR–MAS) is a newer approach to study biological interactions, and HR–MAS NMR has been used for the investigation of the locations and distributions of flavonoids with lipid membranes [97]. By spinning the sample with several KHz at the magic angle (54.73° to the external magnetic field), line broadening effects are removed, resulting in high-quality spectra. The specific HR–MAS probe combines different properties of liquid- and solid-state samples, and MAS allows for acquiring ordinary ^1^H, ^13^C and 2D spectra, similar to solution-state NMR [34,96,97]. The sample preparation can be performed, for example, according to Gréland et al. [98] by dissolving a lipid/polyphenol mixture in D_2_O and subsequently handling it via freeze–thaw in order to form the lipid bilayers. Then, the emulsion is transferred into an specific HR–MAS insert that is placed into the HR–MAS rotor and then in the instrument [34]. ^1^H MAS NMR can be used to measure ring current-induced chemical shift changes: for example, the interactions between flavonoids and phospholipid bilayers cause the lipid signals to slightly shift upfield [97]. These shifts are due to the ring current effect of the delocalized π-electron system of the flavonoid ring structures. The proximity of the lipid protons to the aromatic ring systems affects the magnitudes of the chemical shifts of the lipid signals: the larger the ring current-induced shifts, the stronger the interaction between lipids and polyphenols [97]. The magnitude of the signal shift tells how much the added polyphenol affected the spatial surroundings of the lipid proton, thus indicating how far into the lipid bilayer the polyphenol can penetrate [34,97]. In the presence of flavonoids, the lipid signals of POPC (see Figure 2 for the structure) are slightly shifted to upfield [97]. These results showed that flavonoids were localized and broadly distributed in the lipid membrane, with a maximum in the lipid/water interface, and when the number of OH groups in the flavonoid structures increased, the maximum of this distribution was biased toward the lipid headgroups [97]. Similar observations have been made for HTs and lipid signals of *E*.*coli* [34]. In these studies, the chemical shifts of H-C2, H-*β* and H-G1 (see Figure 4 for the lipid structure) were mostly indicating that the tannins could penetrate the lipid bilayer at least until H-C2.

Another interesting method of studying polyphenol–lipid interactions is nuclear Overhauser effect spectra (NOESY) to see the spatial proton–proton correlations. The cross-relaxation between nuclei is formed during the mixing time. In general, the cross-relaxation is formed faster for large molecules than for small molecules, and for that reason, shorter mixing times are better for large molecules. The distance between the nuclei also affects the cross-relaxation formation. Therefore, different mixing times, for example 0.1 s and 0.3 s [34], are typically used in NOESY experiments. NOESY spectra provide information about the locations and orientations of polyphenols in lipid membranes. The NOESY cross-relaxation rates can be interpreted as contact probabilities between the interacting protons of neighboring molecules [97]. The strength of the polyphenol–lipid interactions can be evaluated based on the cross-relaxation rates: higher rates indicate stronger interactions. From the NOESY spectra, lipid signal volumes (i.e., diagonal peak volume, abs) and their correlation signals to the aromatic protons of polyphenols (i.e., cross-peak volumes, abs) are integrated; then, the cross-relaxation rates can be simply calculated from the NOESY spectra based on the following equation [34,99]:

(2) $$\mathrm{cross}\;\mathrm{relaxation}\;\mathrm{rate}=\frac{\left(\frac{\mathrm{cross}\;\mathrm{peak}\;\mathrm{volume}}{\mathrm{number}\;\mathrm{of}\;\mathrm{cross}\;\mathrm{peak}\;\mathrm{protons}}\right)}{\mathrm{diagonal}\;\mathrm{peak}\;\mathrm{volume}\;\times \mathrm{mixing}\;\mathrm{time}}$$

The other option is to measure the NOESY spectra at various mixing times, integrate the volumes of the respective diagonal and cross-peaks and then fit the results according to the spin pair model yielding more accurate cross-relaxation rates [97,100]. Based on NOESY experiments, Scheidt et al. showed high cross-relaxations rates of the 2´/6´protons of the flavonoids with the protons of POPC lipids and demonstrated that ring B of the flavonols luteolin and myricetin is pointing towards the aqueous phase, while ring A is inserted deeper in the acyl chain region of the lipid membrane (see Figure 1 in this paper for the example structure of flavan-3-ols with ring label letters and Figure 6 in [97] for details about the cross relaxation rates and distributions), whereas for flavone chrysin, an opposite orientation was determined [97]. Virtanen et al. showed with NOESY experiments that tannins exhibited the highest cross-relaxation rates against the lipid protons H-C3, H-C2, H-G1 and H-CH/H-G2, indicating that tannins penetrate the lipid bilayers close to protons of the lipid headgroups [34]. In the case of casuarictin and tellimagrandins I and II, the protons in the galloyl groups seemed to be slightly closer to the protons H-C3, H-C2 and H-G1 (closer to the hydrophobic tail), whereas the protons in the hexahydroxydiphenoyl (HHDP) groups had slightly higher cross-relaxation rates against lipid protons H-G1 and H-CH/H-G2, indicating them to be closer to the hydrophilic head groups (Figure 4).

The results obtained from the shifts in the lipid signals in ^1^H NMR spectra and the correlation signals in NOESY spectra can be supported by the diffusion measurements by pulsed field gradient (PFG) NMR [97]. PFG–NMR utilizes MAS conditions and the stimulated echo sequence with bipolar gradient pulses and eddy current delay before detection as discussed in [97,101,102] and gives the mobility and lateral diffusion of the polyphenols in lipids. In practice, the techniques based on NMR spectroscopy provide detailed data, and the sample preparation is rather straightforward. However, the instrumentation is expensive to purchase and maintain, and its use requires specific know-how; in addition, in-depth knowledge in chemistry and especially in spectroscopy are needed as NMR spectra can be complex with overlapping signals. One limitation to the NMR analysis of polyphenol–lipid interactions is the technical need for rather high polyphenol concentration, which may cause alteration of the lipid bilayer structures or the aggregation of polyphenols [97].

### 2.4. Molecular Dynamics Simulation

Molecular dynamics simulations have become a standard tool for studies of biomolecules in recent years [103]. A search in SciFinder^n^ database with the words “polyphenol” and “molecular dynamics simulation” yields a trend of yearly increasing number of publications, with a most recent total of 147,073 references, indicating that the simulational approaches are growing rapidly [104]. This progress is supported by developments in the computing hardware, algorithms and software that allow for longer and cheaper simulations [105]. In general, the molecular dynamics simulations are powerful tools for elucidating the mechanisms of interactions at the atomic level, complementing the experimental methods such as X-ray crystallography, cryo-electron microscope, NMR, paramagnetic resonance and Förster resonance energy transfer (FRET) [33,103,106]. Molecular dynamics simulations can be used in stand-alone research or to interpret experimental results or guide the experiments, for example, for structural and dynamic studies (to study conformational flexibility and stability), perturbations (to observe response after a controlled change to a system) and processes (to observe a dynamic process over time) [105]. For polyphenol–lipid interactions, these studies have included the penetration, location and orientation of polyphenols in the lipid bilayer [107,108,109] and the effects of the structural features of polyphenols on these interactions [106,110].

The basic idea of molecular dynamics simulation is rather straightforward: to create the molecular system, assign force field parameters and then predict how the particles move. However, this all requires a solid understanding of both the biological system and the theoretical basis of molecular dynamics simulations [105]. First, the positions of all atoms in a biomolecular system, such as polyphenols penetrating lipid bilayers, need to be known. This includes the modeling of pre-equilibrated structures and of the topologies of membranes, 2D chemical structures and 3D conformations of polyphenols. Then, the force exerted on each atom by all the other atoms can be calculated. The forces in molecular dynamics simulation are calculated using a model called a molecular mechanics force field, which is fitted to the results of quantum mechanical calculations and typically also to particular experimental data [105]. The common choices are AMBER (assisted model building with energy refinement), CHARMM (Chemistry at HARvard Macromolecular Mechanics) and OPLS (optimized potentials for liquid simulations), and for phospholipid systems, the Gromacs 2019.1 software with the GROMOS G53A6 force field has been used [106,111]. Community-driven GROMACS [112] is one of the most widely used free and open-source software in chemistry [111] and it is primarily used for high-performance molecular dynamics simulations of biomolecules. The GROMOS G53A6 force field has been commonly used for the molecular dynamics simulation of lipid membranes with sufficient accuracy [106,113], and Zhu et al. used the GROMOS53a6 force field and Berger lipid parameters in studies on the penetration of galloylated and nongalloylated PAs into POPC:1-palmitoyl-2-oleoyl phosphatidylethanolamine (POPE) (1:1) lipid bilayers [110]. However, it has to be remembered that the force fields are approximations even though they have developed significantly in recent years [105]. After the molecular dynamics simulations, the spatial position of each atom as a function of time can be predicted, and repeatedly calculating the forces on each atom and using them to update the position and velocity of the atom, the simulation output, i.e., a trajectory, can be created [105,114]. Then the analysis of trajectories can be carried out by several auxiliary programs provided within the Gromacs 2019.1 software [106]. These analyses can be challenging as simulations produce a huge amount of data and it is not easy to identify the most relevant and biologically important aspects [105]. Molecular dynamic simulations can be combined with binding free energy calculations: the binding free energy can be calculated from the snapshots of molecular dynamics trajectory, for example, using the reliable and commonly used molecular mechanics/Poisson–Boltzmann surface area (MM/PBSA) [106]. As an end note, the molecular dynamics simulation methods are a powerful technique for understanding the biomolecular processes. Improvements in the computational equipment and molecular dynamics algorithms enable the better analysis of conformational ensembles representing the real macromolecule structures and matching with the experimental data [114]. However, molecular dynamics simulation requires fast hardware and in-depth knowledge, so it is not necessarily available for all. Therefore, tools are needed to popularize molecular dynamics simulations as, so far, they have been restricted to research groups that have the expertise needed; in the worst cases, nonexpert users are utilizing default procedures, which may lead to artifactual trajectories that are difficult to separate from the correct ones [114]. In the future, the simulations will become even faster, cheaper and more accurate [105], so the widely accessibility with a simple setup suggested [114] would make them nicely available for all.

## 3. Understanding the Polyphenol–Lipid Interactions

The investigation and understanding of the polyphenol–lipid interactions is a complex process including different model polyphenols, lipids and chemical methods. The intensity of the interactions, the affinity of polyphenols to lipids and the forces included are related to the structure of the polyphenols but also to their locations and orientations in the lipid bilayers. Polyphenols penetrating the lipid bilayer can cause changes in its structure and physicochemical properties.

### 3.1. Location and Orientation of Polyphenols in the Lipid Bilayers

Polyphenols show a large distribution along the lipid membrane depending on the structures of both the polyphenols and the lipid. It is widely accepted that the deeper the permeation of ligand into the lipid bilayer, the higher the affinity between the ligand and the lipid bilayer [106,115]. In many cases, polyphenols are located close to the polar headgroups of the lipids [34,51,106,107,116,117], but in fact, depending on the structures of the polyphenols and the compositions of the membrane lipids, the locations may vary. For example, based on molecular dynamics simulation, diarylheptanoid curcumin stays in the lipid tail region, close to the interface of the lipid head and lipid tail, most probably in parallel orientation relative to the lipid bilayer [29]. Trans-stilbene resveratrol is mainly located in a deeper region of the membrane, interacting with the lipid tails [35,37,118,119], but some studies support its location close to the polar headgroups [120]. Furthermore, simple phenolic oleuropein aglycone is known to be located between the hydrocarbon acyl chains of the phospholipids, although its exact locations and molecular interactions are different in different lipid systems: In the POPC/cholesterol systems, it is closer to the membrane surface, whereas it penetrates more deeply in POPC/1-palmitoyl-2-oleoyl-sn-glycero-3-phosphatidylglycerol (POPG)/cholesterol systems [121]. Similarly, the above-mentioned resveratrol has higher affinities for the more fluid EPC bilayers than for the more organized EPC:cholesterol and EPC:cholesterol:sphingomyelin membrane models [35]. These effects of the polyphenol structures and the compositions of the membrane lipids on the location on the polyphenol within the lipid bilayer account for the variability observed in the studies on polyphenol–lipid interactions and have also been addressed earlier by Reis et al. [122]. External factors and ambient conditions also have effects: the incorporation of polyphenols into lipid bilayers is increased with the increasing salt concentration of the aqueous medium and decreased with the increasing negative electric charge of the lipid membranes [123].

Flavonoids show a large distribution along the membrane, but as the number of OH groups in flavonoids increases; thereby, the hydrophobicity of the flavonoids decreases, and the maximum of this distribution bends toward the lipid headgroups [97]. For example, the B ring of luteolin and myricetin is pointing toward the aqueous phase and the A ring is closer to the acyl chain region, whereas the A ring of chrysin is toward the aqueous phase [97]. See Figure 1 for the example structure of flavan-3-ols with ring label letters. In molecular dynamics simulation studies of the location and orientation of quercetin and its glucuronidated, methylated and sulfated metabolites in lipid bilayers, it was noticed that quercetin and its derivatives penetrate the lipid bilayer to different depths depending on the charge of the molecule and the substitutional variations, i.e., the substituted OH group and the type of the substituent [107]. The interactions of flavan-3-ols and their gallates with lipids have been intensively studied, and they are known to have strong affinities to lipids [19,94,106,123,124,125]. Galloylated flavan-3-ols intercalate within the hydrocarbon chains of the hydrophobic tail, whereas the nongalloylated ones stay in the lipid–water interface [124]. Similar observations have been made for PA dimers: galloylated PA dimers have higher affinities to the POPC/POPE lipid bilayer and penetrate more deeply into the bilayer than the nongalloylated PA dimers [110]. The trajectories of molecular dynamics simulations provide insights into the mobility of polyphenols; for example, with flavan-3-ols, after the absorption into the bilayer, their mobilities substantially decrease; at the end, flavan-3-ols are completely enclosed by the lipid headgroups, and their displacements become restricted [125].

The forces stabilizing the location and orientation of polyphenol in the lipid bilayer are mainly hydrogen bonds. Polyphenols have the ability to form hydrogen bonds between their hydroxyl groups (H-bond-donating moieties) and the C=O and phosphate groups of polar lipid headgroups (H-bond-accepting group) [99,106,125,126]. The degree of hydrogen bonding is affected by the polyphenol structures and as well as the locations: Fewer hydrogen bonds are formed for the flavan-3-ol epigallocatechingallate (EGCG) than for epigallocatechin (EGC) even though the number of hydroxyls is greater in EGCG [106]. This might be because EGCG penetrates more deeply into the lipid and is located at the acyl chain region, which can increase the distance between the hydrogen donor and the hydrogen acceptor [106]. In addition to hydrogen bonding, the location and orientation of polyphenol can be stabilized by van der Waals interactions [106]. The flavonoid–lipid interactions can involve charge–dipole and dipole–dipole interactions as flavonoids have relatively strong dipole moment between 3.6 and 6.6 D for the molecules studied [97].

### 3.2. Effects of the Structural Features of Polyphenols

**Figure 5:**
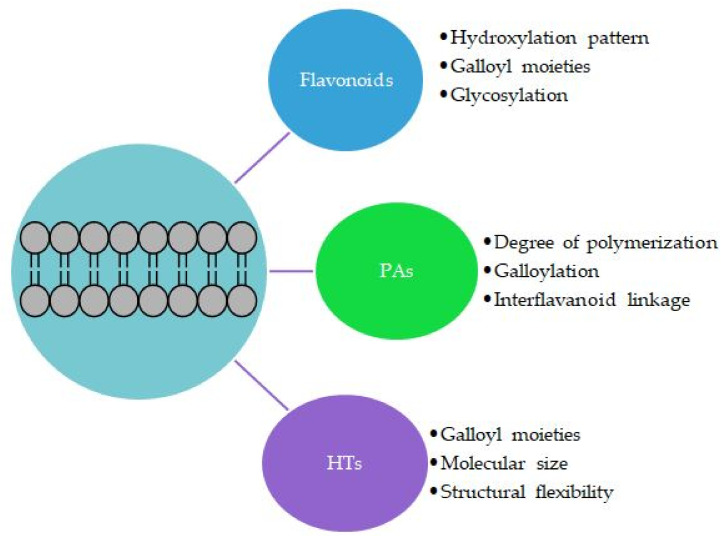
Main structural features of polyphenols affecting their interactions with the lipid bilayers; PAs = proanthocyanidins, HTs = hydrolysable tannins. In addition, the physicochemical properties, such as hydrophobicity and dipole moment, of the polyphenol, the lipid composition and intermolecular forces affect.

The ability of polyphenols to permeate the lipid bilayer depends on their physicochemical and structural properties (Figure 5). The hydrophobicity of the polyphenol is important [19,29,47,82,94,97,127], but it does not fully determine its ability to penetrate the lipid bilayers. For example, the fairly hydrophobic ellagitannins geraniin, chebulagic acid and chebulinic acid show only very weak affinity for lipid vesicles determined by ITC [29]. For flavonoids, the hydroxylation pattern of their tricyclic skeleton affects mostly the orientations of the flavonoids in the lipid bilayer, as discussed above in Section 3.1. Even one OH group can make a difference in the affinity of flavonoids to lipids, as evidenced by membrane effects of kaempferol and quercetin [117]. The highest lipid affinity of flavonoids is related to the 3-hydroxylation of the heterocyclic C ring, which makes flavonols more active than the structurally corresponding flavones [128]. In addition, a nonmodified B ring or a decreasing number of OH groups in the B ring increases the affinity of flavonoid to lipids; for example, galangin, which has no OH group in the B ring, shows very strong membrane activity [128]. The affinity also increases with the 5,7-dihydroxylation of the A ring followed by the 3′,4′ -dihydroxylation of the B ring [128]. In the studies on the interactions between flavonoids and DOPC monolayers, clear structure activity was observed [40]. For flavonoid aglycones, quercetin was the most active, then kaempferol > naringenin > hesperetin > catechin, and for flavonoid glycosides, tiliroside > rutin > naringin; in general, the flavonoid glycosides with two glycose moieties interacted less with the lipids as the sugar moieties made the molecules less hydrophobic and larger in size [40]. In the case of the citrus flavonoids hesperidin and naringin, it was noticed that even a small structural difference in the flavonoid structures, for instance, naringin contains one additional methoxy group compared with hesperidin, can influence the membrane bilayers distinctly [77]. In general, the methylation increases the hydrophobicity of flavonoids, as shown for genistein and kaempferol [129].

The galloyl group seems to be vital in the membrane interactions of polyphenols: the more galloyl groups there are in the structure, the more lipophilic is the polyphenol and the deeper it is in the lipid bilayer [124]. Flavan-3-ol gallates penetrate more deeply in the hydrophobic acyl chain region in the lipid bilayer than the ones without gallate [106,125,126]. For example, EGCG has the ability to form hydrogen bonds with the deeper inside oxygen atoms in the POPG lipid bilayer, and the galloyl moiety seem to be the key functional group for EGCG to form hydrogen bonds with the POPG lipid bilayer in comparison with EGC [106]. With tea flavan-3-ols, epicatechingallate (ECG) has the highest affinity for lipid bilayers, followed by EGCG and epicatechin (EC); no EGC was detected in the lipid bilayer [19]. This order also supports the observations by Tsuchiya [128] that decreasing number of OH groups in the B ring increases the affinity of flavonoid to lipids as ECG and EC have the 3′,4′ -dihydroxylation of the B ring, whereas EGCG and EGC have the 3′,4′,5′ -trihydroxylation of the B ring. The partition coefficients of flavan-3-ols in n-octanol/PBS decreased in the same order [19]. The degree of hydroxylation is linked to the hydrophobicity of the flavonoids: When the number of OH groups increases, then the hydrophobicity of flavonoids decreases [97,127], both reflecting the affinities of flavan-3-ols for the lipid bilayer.

Similar observation has been made for galloylated PAs: Zhu et al. showed that the galloylated A-type procyanidin and prodelphinidin dimers have much higher affinities to the lipid bilayer with lower binding free energies in comparison with nongalloylated PA dimers, indicating that the gallates also play a vital role in the membrane interactions of PA dimers [110]. There are three possible explanations for the vital role of the galloyl group [110]: (1) Galloyl moieties provide more efficient OH donors for the hydrogen bonding and thereby enhance the binding with the lipid bilayer, (2) galloyl moieties increase the hydrophobicity of the molecules [45,48] and thereby enhance the binding in the hydrophobic region of the lipid bilayer, or (3) galloyl moieties affect the spatial configuration of the PA dimers, allowing the main skeleton of the PA dimer to outstretch on the surface and the galloyls to permeate more deeply inside of the lipid bilayer, which leads to more efficient contact between H-bond donors and acceptors [110]. The other structural features of oligomeric and polymeric PAs affecting their interactions with the lipids are the degree of polymerization and the type of interflavanoid linkage [130,131]. Based on the molecular dynamic simulation, Zhu et al. concluded that the position and orientation of PAs in lipid bilayers depend on both their degree of polymerization and the interflavanoid linkage type: The depth of the penetration decreased with the increase in the degree of the polymerization, and A-type PAs formed more hydrogen bonds with the deep oxygen atoms in the lipid bilayer [132]. For example, persimmon PAs are highly polymerized, with a mean degree of polymerization of 26, and also highly galloylated (72%), and these two features have been concluded to be the reason for their noticeable biological activities [130]. For HTs, in addition to the galloyl moiety, the structural flexibility of the entire tannin structure and the molecular size are important [29].

### 3.3. Changes to Lipids and Their Physical Properties

When polyphenols penetrate the membrane lipids, they can perturb the lipid bilayers and their properties in many different ways. They can, for example, increase or decrease the acyl chain order, fluidity or rigidity of the lipid membrane. The changes detected in the lipid order vary depending on the polyphenol and lipid studied. For example, the studies on the interactions of kaempferol and quercetin with the membranes of EPC:sphingomyelin (2.4:1) and of 1,2-dipalmitoyl-*sn*-glycero-3-phosphocholine have shown that these flavonoids increase the lipid order [117]. For hesperidin, a weakly disordering effect has been detected in the hydrophobic region of the lipid membrane, while naringin has an ordering effect in this region; however, both flavonoids induce alterations in the arrangement of the polar heads of lipids [77]. In a study utilizing experimental DSC, steady-state fluorescence anisotropy, fluorescence spectroscopy and FTIR, the flavan-3-ols catechin, EC, ECG and EGCG were reported to affect the physical properties of phospholipid membranes and increase the lipid order with tightly packed acyl chains [124]. On the other hand, EGC, ECG and EGCG on the surfaces of lipid bilayers have been found to perturb the membrane structure [19,106]. EGCG is also known to penetrate more deeply into the POPG lipid bilayer than EGC, thereby possessing more potent structure-perturbing potency than EGC [106]. Using molecular dynamics simulation, ECG and EGCG were found to change the lipid bilayer structures by increasing the area per lipid and decreasing the lipid bilayer thickness as well as the lateral diffusion coefficients of the lipids [106]. EGCG and EGC also caused the attenuation of lipid acyl chains alignment, which might result in the more disordered membrane structure [106]. Similar findings were observed for the galloylated PAs in molecular dynamics simulations: the galloylated PAs induced the strong lateral expansion of the POPC/POPE (1:1) membrane and thereby increased the disorder of the lipid tails [110].

Cell membrane fluidity has an important role in cell physiology. Several polyphenols have been reported to affect the cell membrane fluidity and thereby contribute to the cell homeostasis [37,89,128,130,131]. The increased fluidity of the lipid membrane has been observed, for instance, for tea flavan-3-ols disturbing the membrane structure [133], for kaempferol and quercetin facilitating the penetration of water molecules into the membranes [117] and for trans-stilbene resveratrol and its glucoside piceid changing the membrane properties, increasing fluidity and inducing fluctuation [118]. However, for the rosmarinic acid–membrane interactions, no significant modification of the membrane permeability and fluidity was observed [116]. The effect on the cell membrane fluidity can also be dependent on the polyphenol concentration depending on the hydrophobic/hydrophilic character of the substances: kaempferol decreases the membrane fluidity at low concentrations, but increases it at high concentrations [117]. Similarly, the effect of quercetin on the fluidity and local order of the lipid membranes depends on the concentration, whereas the effect of EGCG is not dose-dependent [89]. The polyphenols can also cause membrane aggregation and rigidification through their interactions with the membrane lipids. This phenomenon has been reported at least for flavonoids [118,128].

## 4. Conclusions

Polyphenol–lipid interactions are of great importance as the affinity of polyphenols for lipid membranes affects their biological activity. Generally speaking, for high affinity to lipids, two factors are important: First, the hydrophobicity of the polyphenol matters: More hydrophobic polyphenols can penetrate more deeply into the lipid bilayers, and second, the forces stabilizing the location and orientation of the polyphenol in the lipid bilayer are important. These two things are dependent on the structures of the polyphenols and the compositions of the membrane lipids. The polyphenol–lipid interactions can be studied in detail at the molecular level using the state-of-the-art techniques such as HR–MAS NMR spectroscopy and molecular dynamics simulation. These techniques provide comprehensive information about the locations, orientations, mobility and lateral diffusion of polyphenols within the lipid bilayers. However, these techniques are not readily available for everyone, and their usage requires a notable amount of prior knowledge and expertise. Therefore, in some applications, partition coefficient measurements using octanol–water or membrane models provide feasible results for evaluating these interactions: in general, the hydrophobicity of the polyphenol predicts its ability penetrate the lipid bilayers. However, as discussed above, the hydrophobicity of the polyphenol does not fully determine its affinity to lipids.

In general, there is still room for improvements in chemical methodology. Future computational chemistry can offer even better possibilities for studying these interactions. Multiple models with various force fields can be utilized in order to make sure that most of the physicochemical characteristics are correct; for example, the conformational flexibility and steric effects of tannins make their analysis challenging. There exists a risk that the computational methods produce unnecessary data without in-depth knowledge. However, this risk can be reduced by producing a considerable amount of specific, high-quality experimental data, for example by HR–MAS NMR, to support the calculations. Polyphenol–lipid interactions are complex and challenging to study, and the complexity is increased even further if the other macromolecules and components in the biological system are considered. The interactions of all components might be, for instance, distinct, synergistic, antagonistic or competing. There seem to be similar structural features of polyphenols affecting their interactions with proteins and with lipids; for example, the molecular size, conformational mobility and flexibility of tannins are important in their macromolecule interactions. Similarly, the methylation of flavonoids increases their hydrophobicity but also their affinities for proteins, and different polyphenols have different affinities for proteins, carbohydrates and lipids. Can we in the end conclude an affinity order of polyphenols for different macromolecules? What are the combined effects resulting from the different interactions between polyphenols and macromolecules? Can we even generalize the interactions or are they so highly specific that the overall view is not possible? With systematic further studies using well-known model substances and several different methods, we can find answers to these questions and better understand the biological context of these interactions.

## Data Availability

Not applicable.
